# Aqueous dispersions of oxygen nanobubbles for potential application in inhalation therapy

**DOI:** 10.1038/s41598-022-16720-3

**Published:** 2022-07-21

**Authors:** Marcin Odziomek, Karol Ulatowski, Katarzyna Dobrowolska, Izabela Górniak, Paweł Sobieszuk, Tomasz R. Sosnowski

**Affiliations:** Faculty of Chemical and Process Engineering, Warsaw University of Technology, Waryńskiego 1 Street, 00-645 Warsaw, Poland

**Keywords:** Drug delivery, Respiratory tract diseases, Nanoscale biophysics, Drug delivery

## Abstract

Inhalation is a non-invasive method of local drug delivery to the respiratory system. This study analyzed the potential use of aqueous dispersion of oxygen nanobubbles (ADON) as a drug carrier with the additional function of oxygen supplementation to diseased lungs. The suitability of the membrane-based method of ADON preparation and, next, the stability of ADON properties during storage and after aerosolization in nebulizers of various designs (jet, ultrasonic, and two vibrating mesh devices) was investigated. The increased oxygen content in the aerosol generated in two mesh nebulizers suggests that the proposed concept may be helpful in the oxygen supplementation during drug delivery by aerosol inhalation without using an additional oxygen source. This application can increase the overall effectiveness of lung disease treatment and pulmonary rehabilitation.

## Introduction

Nanobubbles (NBs) are one of the most significant recent developments in gas–liquid systems^1,2^. Most commonly, NBs are spherical gas objects in liquid with a diameter below 1 μm^3–5^. Their applications in the multiple branches of science and industry are gaining worldwide attention^2,6–11^. NBs have several medical and therapeutic applications. Aqueous dispersions of nanobubbles are used in chronic wound treatment^12^, and oxygen nanobubbles with surfactant shells are used as the ultrasonographic contrast^13–15^. Some novel applications are also considered^9,16^, including hypoxia treatment by intravascular delivery of oxygen NB in COVID-19 patients^17^.

Pulmonary diseases and dysfunctions are widely treated with inhalations which allow a direct deposition of medicines on the surface of the respiratory system^18,19^. This method is used to deliver bronchodilators (e.g., short- of long-acting beta-agonists) and anti-inflammatory agents (e.g., glucocorticosteroids), which are the primary therapeutics in asthma and COPD, but also COVID-19^20–22^. Inhalation of aerosols formed by atomization of liquid medicines (solutions or suspensions), known as nebulization, can be conveniently used in poorly cooperating patients, such as disabled or even unconscious patients, and infants or toddlers. Drug delivery by this technique is always possible since it does not require any special breathing maneuvers compared to using other inhalers (pMDIs, DPIs)^23^. Many pulmonary diseases are related to impaired gas exchange, so oxygen therapy is often required to treat severe dysfunctions of the respiratory system. Oxygen delivery can be joined with drug inhalation producing a synergic effect^24^. Increased oxygen delivery without inhaled medicines is also helpful in pulmonary rehabilitation during stable disease^25^, or the recovery phase^26^.

Considering the above, aqueous dispersion of oxygen nanobubbles (ADON) can be proposed as vehicles of drugs delivered by inhalation of aerosols from nebulizers. The application of NBs in inhalation therapies can enhance the therapeutic effect in two ways. The modified liquid structure by a high interfacial area of NBs can facilitate the transport of the drug to lung surface and to the cells. At the same time, NBs increase the oxygen supply to the respiratory system, which seems particularly important in the face of the ongoing SARS-CoV-2 pandemic and its severe consequences, such as pediatric inflammatory multisystem syndrome (PIMS) and post-acute COVID-19 syndrome^27^. An additional potential advantage of inhalation therapies with ADON is that patients, in most cases, can carry out inhalations themselves using medicines available in the pharmacy stores. The essential condition is the stability of nanobubbles in the dispersion, which have been already confirmed in several systems^1,3^ but still require additional proof in this particular application.

This study was focused on several basic aspects required to consider ADON as potential solvent of nebulized drugs, i.e.:

1. the suitability of NB generation method and the stability of ADON during storage, including the conservation of density of NB size distribution and oxygen concentration,
2. the influence of NBs on the nebulization process in different nebulizers, including aerosol droplet size and fine particle fraction (FPF),
3. the influence of nebulization process on NBs stability and oxygen concentration in the aerosol.

## Methods

### ADON preparation and stability testing

**Figure 1:**
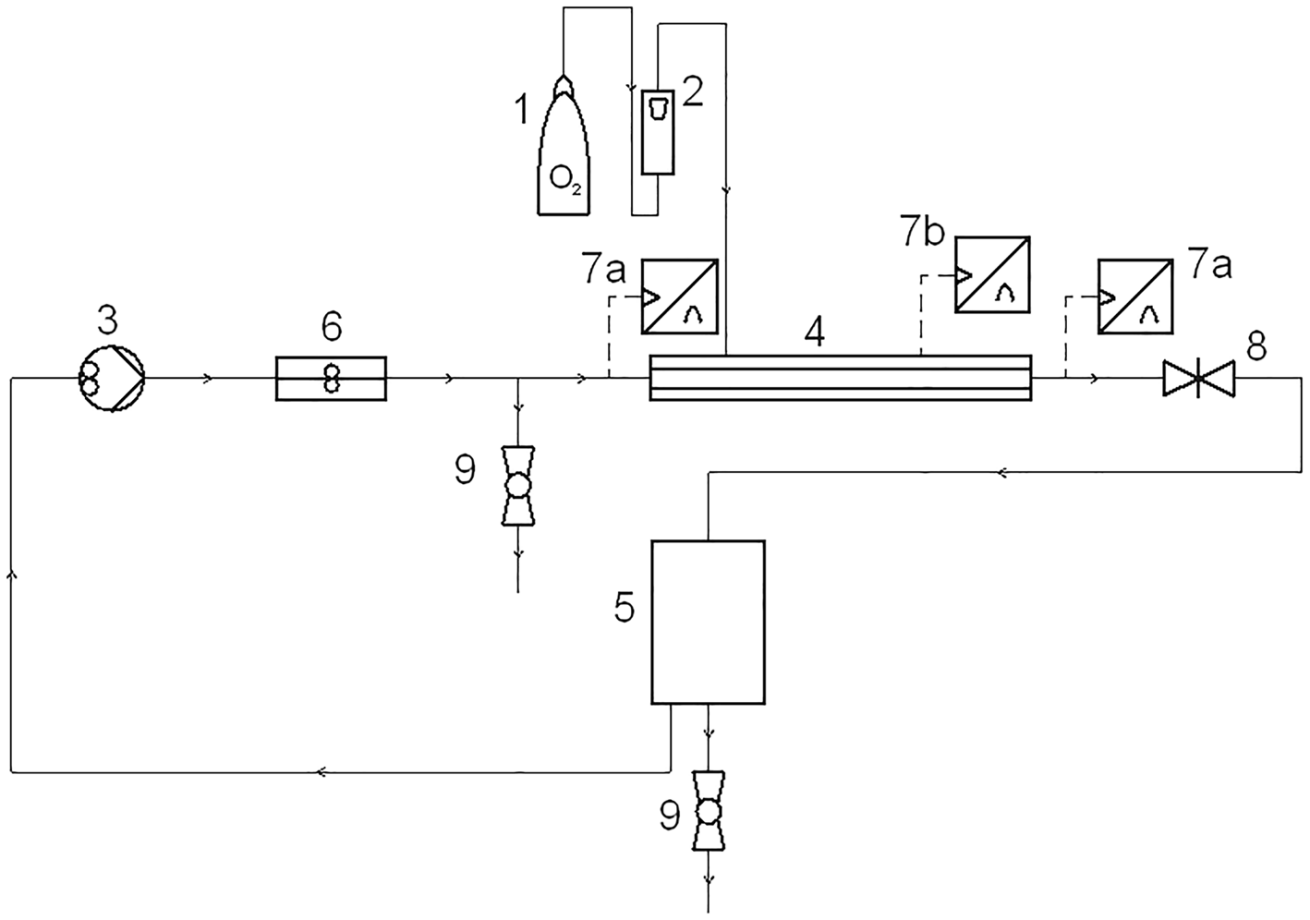
Nanobubble generation setup (made by Fine Bubble Technologies, Poland). (1) oxygen cylinder, (2) gas rotameter, (3) gear pump, (4) module with ceramic membrane, (5) tank for nanodispersion, (6) turbine liquid flow meter (Kobold Instruments, Poland), (7) pressure transducer UPT20 (WIKA, Poland) for: (a) liquid, (b) gas, (8) gate valve, (9) ball valve.

ADONs were prepared in generation setup (Fig. 1) with cylindrical porous ceramic membrane (ZrO_2_ on TiO_2_ support, SiC membrane pore diameter 0.14 μm, internal/external membrane diameter: 8/10 mm, membrane length 125 mm; Tami Industries, France). Membrane was enclosed in stainless steel casing which allowed for gas supply to the membrane in controlled manner. Gas was able to freely fill whole volume of the casing. During the generation process, the pressurized oxygen from the cylinder was forced through the membrane and the shear stress of the flowing distilled water caused the nanobubbles to be detached from the membrane surface. Nanodispersion was recirculated in the setup and stored in the 5 L stainless steel tank. Generation was carried out in 4 L of distilled water for 30 min with constant gas pressure and volumetric flowrates of liquid (*Q*_*l*_) and oxygen (*Q*_*g*_) (Table 1). Liquid pressure drop in membrane module ΔP was calculated as the difference between indications of two pressure transducers set on the liquid path (denoted as 7a in Fig. 1). These conditions were selected as optimal after preliminary tests using different parameters *Q*_*g*_, and *Q*_*l*_ (data not shown). Samples were gathered to the plastic containers (for immediate usage) or to the glass vials which were closed and secured with parafilm (for storage/usage after prolonged time). Samples were then used to measure the quality of the dispersion (density of nanobubble size distribution, Sauter diameter, oxygen concentration in liquid) and to determine the characteristics of atomization process carried out in the nebulization chambers. ADON stability was tested after 1, 4, 7, 14 and 21 days of storage.

**Table 1:** Parameters of ADON generation process.

Gas pressure *P*_*g*_ (bar)	Liquid pressure *P*_*l*_ (bar)	Liquid pressure drop in membrane module *ΔP* (bar)	Gas flowrate *Q*_*g*_ (mL min^−1^)	Liquid flowrate *Q*_*l*_ (L min^−1^)
3.0	2.7	0.5	6.4	6.9

### Density of nanobubble size distribution, Sauter diameter and zeta potential

Density of NB size distribution was determined by Dynamic Light Scattering method (DLS) using Zetasizer NanoZS (Malvern Panalytical, Malvern, UK). Size distribution was additionally characterized by Sauter mean diameter *d*_32_:

1 $${d}_{32}=\frac{\sum {n}_{i}{d}_{i}^{3}}{\sum {n}_{i}{d}_{i}^{2}}$$

where *n*_*i*_ is the number fraction of bubbles with diameter *d*_*i*_. Sauter mean diameter is typically used in the analysis of fluid dynamics and mass transfer processes.

This type of measurements was done for ADONs: freshly prepared (30 s after sampling from the generation system), after different periods of storage, and in the liquid samples collected from nebulized aerosols (see “Nebulization and aerosol collection” section).

Once for each generation charge, the zeta potential was assessed using Zetasizer NanoZS along with dip cell for microelectrophoretic measurement.

### Nebulization and aerosol collection

Four types of medical nebulizers with different construction and mechanisms of aerosol generation were selected to atomize ADONs. They are described in Table 2.

**Table 2:** Nebulizers used in the studies.

Brand name and manufacturer	Type	Remarks	Designation in the studies
Pari Turboboy SX (Pari GmbH, Germany)	Jet nebulizer	LC sprint nebulizing head	Pari
Thomex MB (Medbryt, Poland)	Ultrasonic nebulizer		Thomex
Aerogen Solo (Aerogen, Ireland)	Vibrating mesh nebulizer (VMN)	Metal mesh	Aerogen
Intec Twister Mesh NE-105 (Intec Medical, Poland)	Vibrating mesh nebulizer (VMN)	Polymeric mesh	Intec

Aerosols formed in the nebulizers were condensed and collected in glass vessels, and then immediately tested for density of NB size distribution. The experiments were done in triplicate. Oxygen concentration measurements were done for distilled water (as a reference) and ADON.

Studied nebulizers had different principles of operation which can help to explain later why not all of them can be suitable for delivery of aerosols formed from ADON. Jet nebulizer consists of a nebulizer head equipped with a nozzle and requires a source of compressed air delivered from the electric compressor. The aerosol is generated in a Venturi-type nozzle in the head and is splashed against an inner baffle which separates large droplets. This causes the partial drainage of liquid and its recirculation, which extends the residence time of the drug inside the vessel. Only small droplets are carried outside the head with a stream of air delivered from the compressor.

Ultrasonic nebulizers consist of a nebulization chamber positioned above a piezoelectric crystal which generates ultrasound with a frequency of 1–3 MHz. The nebulization chamber of the nebulizer used in this study is filled with water and equipped with a medicine cup partially immersed in water preventing overheating the drug during atomization. The drug droplets are torn off the acoustic fountain surface and carried away with the auxiliary airflow. The oversized drops are retained on the impaction baffles and returned to the cup, and only fine droplets forming the inhalable mist is formed can be inhaled by a patient.

In vibrating mesh nebulizers (VMNs), a liquid drug is atomized as it passes through a metal or plastic membrane with micrometric pores made precisely by laser processing. The piezoelectric crystal induces vibrations at the frequency 100–180 kHz. The liquid is pushed through the pores, and liquid fragments are torn off the from the mesh surface forming few-micrometer-size droplets. It is worth noting that the time of liquid conversion to aerosol in VMNs is very short and does not require auxiliary air.

### Aerosol characteristics

Droplet size distribution (DSD) in mists generated in the nebulizers were determined using Spraytec laser diffraction aerosol spectrometer (Malvern Instruments, UK). The device was equipped with 300 mm detector lens and allowed to measure the volumetric size distribution of droplets in the range of 0.1–900 µm. Measurements were done in the time mode (30 s) and rapid data acquisition rate (100 Hz). The raw data were averaged across the measuring time-range during the stable phase of aerosol emission (i.e., for relatively constant values of laser light obscuration and the measured droplet diameter*)*. As the final indicators of aerosol quality, the median volumetric diameter (*Dv50*), geometric standard deviation (*GSD*), and mass fraction of droplets smaller than 5 µm (fine particle fraction—*FPF*) have been determined based on the complete DSD. Respirable particles are typically defined based on mass median aerodynamic diameter (MMAD). However, under certain conditions, Dv50 can be considered as an MMAD equivalent. We used Dv50 because the density of aqueous dispersions is app. equal to 1 g/mL, and the form of droplets released from nebulizers is close to spherical^28,29^.

### Determination of oxygen concentration

Oxygen concentration was measured using optical sensor ProSolo (YSI, USA) which also determines the temperature and surrounding pressure. Two types of measurements were done:

- oxygen concentration determination in ADONs directly either after NB generation or after nebulization and liquid collection (it required collection of samples for 30–40 min);
- oxygen content in aerosol phase formed by nebulized liquids (on-line measurement). In this the sensor was positioned near the nebulizer outlet enabling the complete immersion of the sensor in the freshly formed aerosol. Measurements were carried out until the readings were stabilized (steady-state conditions).

## Results

### ADON properties after preparation and storage

**Figure 2:**
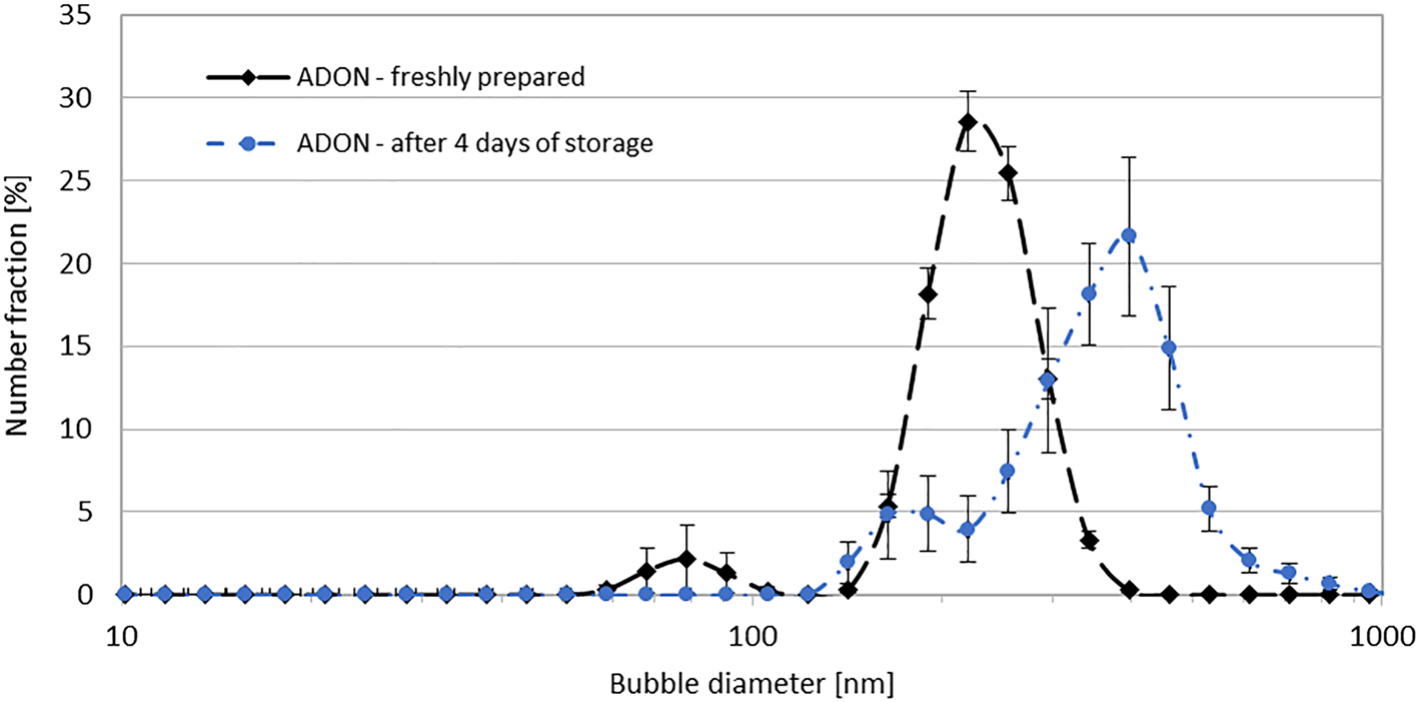
Densities of number size distributions of nanobubbles directly after generation and after 4 days of storage. *ADON* aqueous dispersions of oxygen nanobubbles.

Nanobubble dispersions generated in the porous-membrane system (Fig. 1) were characterized regarding NB size and oxygen concentration. Figure 2 presents the densities of number size distributions of NBs in two ADON samples: immediately after NB generation and after 24 h of storage in a sealed glass vessel at room temperature. The results show that 1-day storage of ADON in the sealed glass vessel allows to preserve oxygen nanobubbles, however their density of size distribution becomes narrower, and the mode diameter increases from ~ 140 to ~ 160 nm. It suggests that ADON undergoes equilibration with some coalescence of NBs. This agrees with results from our previous studies^3^, although ADONs were stored then in plastic containers with a shallow layer of air below the lid, which might lead to partial oxygen desorption to this layer but also through the polymeric walls of the container. These effects additionally explained the change in the densities of bubble size distributions in those studies, however they are absent when ADON is stored in sealed glass vessels without air layer. Additionally, zeta potential of nanobubble dispersions (− 22 mV) was also in agreement with both our previous results and literature references^3,11,30^.

Assuming that ADON for medical purposes medicines needs to be preserved in securely sealed containers, we have checked whether the storage in gas-proof glass bottles affects the size and oxygen concentration of nanobubble dispersions. Table 3 presents the oxygen concentrations and NB Sauter diameters in dispersions directly after generation and after set time intervals. What is worth noting that directly after NB generation (i.e., after 30 s needed for taking the sample and conducting the measurement) ADON is close to complete saturation with oxygen at the given temperature (35.08 ± 2.87 mg L^−1^ of complete saturation). Oxygen content in ADON directly after NB generation is 4.5-fold higher than the equilibrium value expected from physical solubility of oxygen contained in the air in water under these conditions of temperature and pressure. After initial decrease of oxygen content on day 4 to the value 2.3-fold higher than the equilibrium value, oxygen level remains constant until 21 day (when the stability studies were finished), confirming good stability of ADON as an oxygen carrier. We have performed post-hoc Tukey test and it shows that oxygen concentration in nanodispersion directly after generation is significantly different (for $\alpha = 0.001$) from oxygen concentrations in following time points, while from 4th day the oxygen concentration does not significantly change even for $\alpha = 0.05$. NBs are preserved and their Sauter diameter increases from 251 ± 12 nm to 421 ± 73 nm after 4 days and remains at this elevated level (i.e., between 327 ± 73 nm and 454 ± 143 nm) during next weeks. Similarly to oxygen concentration, according to post-hoc Tukey test, the Sauter diameter of bubbles also was significantly different ($\alpha = 0.05$) between nanodispersion directly after generation and after storage in closed glass bottles, while the measurements in following days were not significantly different from one another for the same value of $\alpha$. The only outlier was the Sauter diameter of bubbles after 2 weeks which while still not being significantly different from samples taken after 4 days, 1 week and 3 weeks after generation, was also not significantly different from freshly generated nanodispersion. However, for $\alpha = 0.1$, the significant difference is present. These results are extremely important in the designing any therapeutics for administration after prolonged time from the preparation.

**Table 3:** Properties of ADONs during storage in closed glass containers (room temperature, atmospheric pressure), values are means ± SD, n = 3.

Period of storage	Oxygen concentration (mg L^−1^)	Oxygen content in relation to equilibrium with air (%)	Sauter mean diameter of NBs (nm)
After generation	35.08 ± 2.87	454.58 ± 13.52	251 ± 12
4 days	20.30 ± 0.14	230.80 ± 1.56*	421 ± 73*
7 days	20.90 ± 0.16	241.40 ± 1.90*	432 ± 111*
14 days	20.43 ± 0.09	229.80 ± 0.99*	327 ± 73**
21 days	20.56 ± 0.13	237.50 ± 1.54*	454 ± 143*

Samples denoted with single asterisk are significantly different from sample taken directly after generation with $\alpha = 0.05$, while double asterisk denoted sample significantly different from sample taken directly after generation with $\alpha = 0.1$.

### Aerosol characteristics

**Figure 3:**
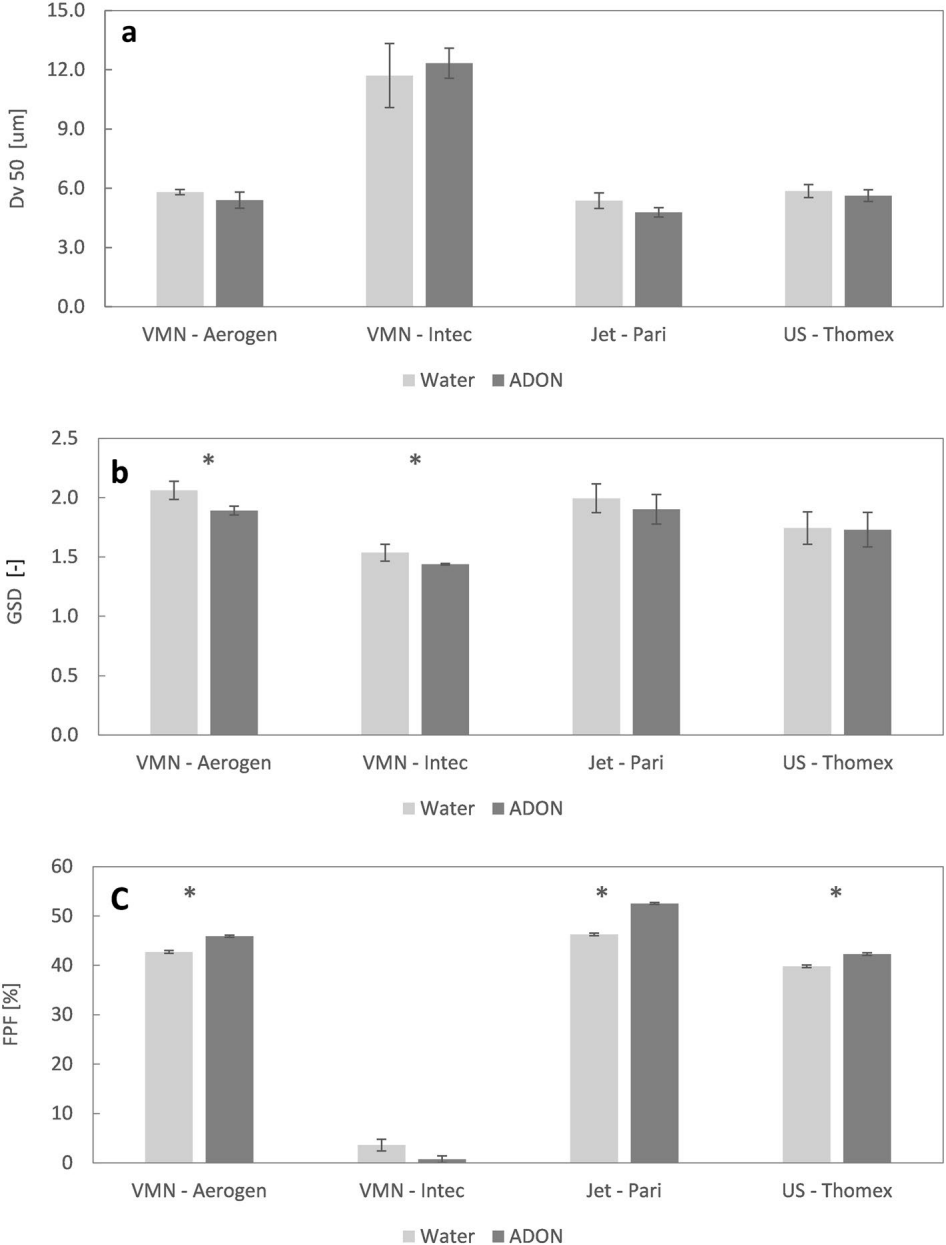
Properties of ADONs and water mists emitted from selected nebulizers: (**a**) *Dv50*, (**b**) GSD, (**c**) FPF. Mean values (n = 3) + standard deviation (error bars). Single asterisks denote the statistically significant differences between samples for the same nebulizer for $\alpha =0.05$.

Figure 3 shows the parameters characterizing aerosols generated in each nebulizer from water and ADON, i.e., the volume median droplet diameter (*Dv50*—Fig. 3a), geometric standard deviation (GSD—Fig. 3b), and fine particle fraction (FPF—Fig. 3c). Three nebulizers generate aerosol with similar *Dv50* (4.5–6 µm), degree of polydispersity (GSD = 1.75–2) and fraction of fine droplets (FPF = 40–50%). In contrast, VMN Intec produces a mist with significantly different properties (*Dv50* > 12 µm, FPF < 10%), suggesting that it is more suitable for treatment of upper airways diseases, such as laryngotracheobronchitis (croup) or other oropharyngeal infections.

Considering the influence of NBs on the properties of produced aerosol, it may be noted that ADON nebulized in three devices: Aerogen, Pari and Thomex, is characterized by slightly lower *Dv50* and increased FPF compared to nebulized distilled water. The most significant change is found for Pari where FPF increased from 45% for water to 52% for ADON. The opposite effect is seen for Intec with slightly increased *Dv50* and FPF reduced from 3% (water) to 1% (ADON), although this parameter is of less importance if aerosol targets the upper airways. In most cases, the difference between aerosolized water and ADON is not statistically significant (see asterisks in Fig. 3), which suggests that ADON can be effectively atomized in various types of commercially available nebulizers allowing generating aerosols suitable for drug delivery by inhalation. As the FPF is significantly larger for three out of four nebulizers, we can assume that presence of nanobubbles in nebulized water allows for easier formation of smaller droplets for most of nebulization mechanisms.

**Figure 4:**
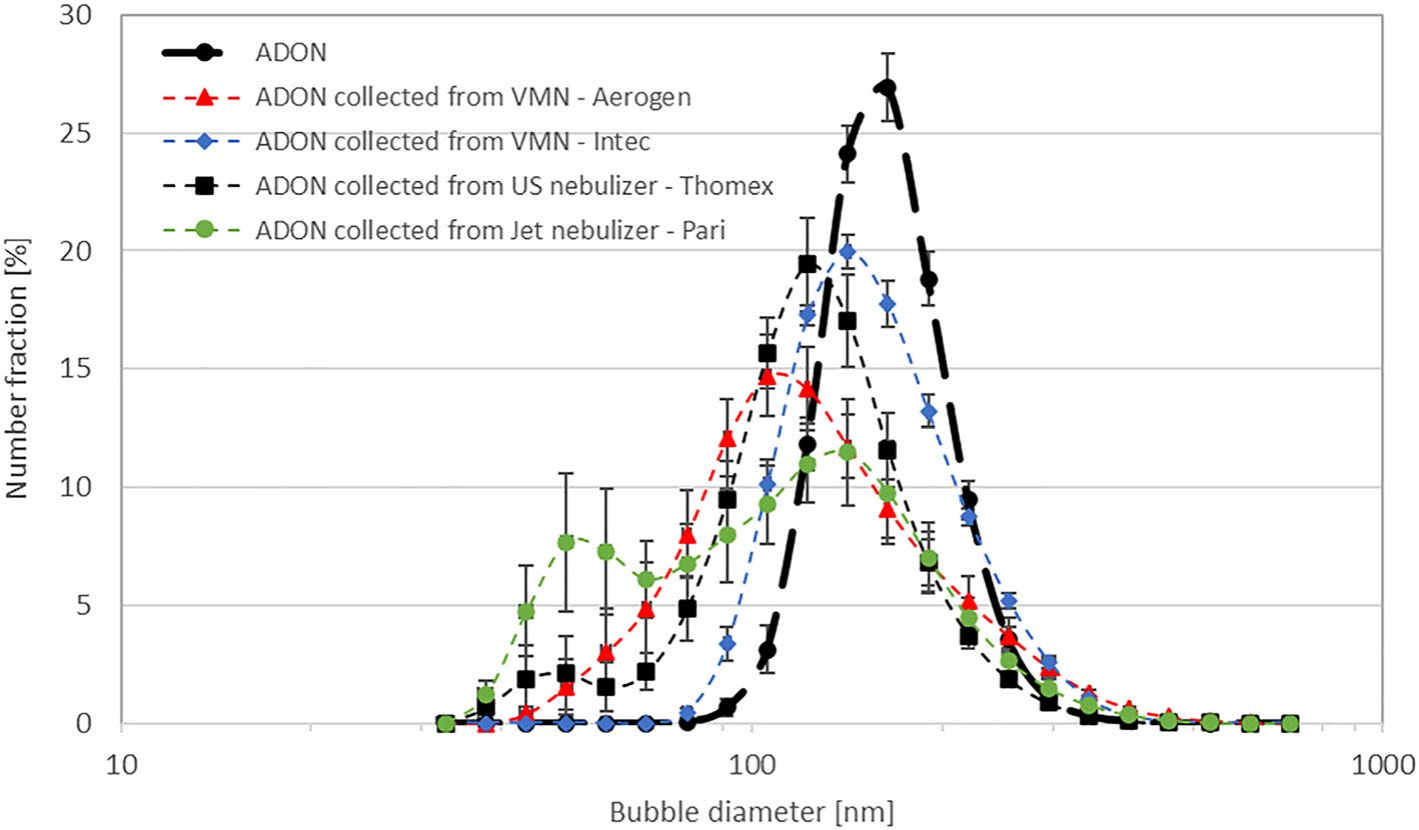
The comparison of densities of number size distributions of nanobubbles before and after nebulization in different nebulizers: vibrating mesh nebulizers (VMN)—Aerogen, and Intec, ultrasonic (US)—Thomex, Jet—Pari.

Next step was to check whether size of nanobubbles is affected by the method of nebulization. For that, measurements of the density of bubble size distributions were done for ADONs after nebulization. As shown in Fig. 4, NBs are still present in the liquids collected from condensing aerosols of nebulized ADONs regardless of the nebulizer type. Simultaneously, there are noticeable differences in densities of size distributions of oxygen NBs present in original and collected dispersions. The mode of the distribution is shifted towards smaller diameters in all collected samples. However, in the case of Pari and Thomex nebulizers we can see an additional peak at bubble sizes below 70 nm, i.e., the distribution changes from unimodal to bimodal. It can be explained by the fact that ultrasonic nebulization affects the density of size distribution of nanobubbles, as ultrasonic waves are able to both destroy and generate new nanobubbles in liquid depending on the process parameters^31,32^. For the jet nebulizer, destruction of nanobubbles may be caused by shear stresses, droplet impaction and liquid recirculation inside the nebulizing head. The least change in nanobubble sizes is observed for Intec vibrating mesh nebulizer, where the change in the mode of nanobubble size decreases from about 180 to 150 nm with a slight increase in the peak width. It may be explained by larger size of droplets obtained in Intec nebulizer, which are formed at lower energy densities and shear stresses. In case of Aerogen vibrating mesh nebulizer, the density of bubble size distribution after atomization is wider than in the initial samples, however the monomodal characteristic is preserved.

Taking into account the changes in density of NB size distribution, we focused next only on two VMNs, i.e., Aerogen and Intec, which caused the least changes in ADON quality after nebulization.

### Oxygen content in nebulized ADON

Knowing that ADON after nebulization contains nanobubbles, this part of the study was intended to check an increase of oxygen concentration in ADON aerosol. The effect of NB presence is assumed to enhance the therapeutic effect of aerosol inhalation.

**Figure 5:**
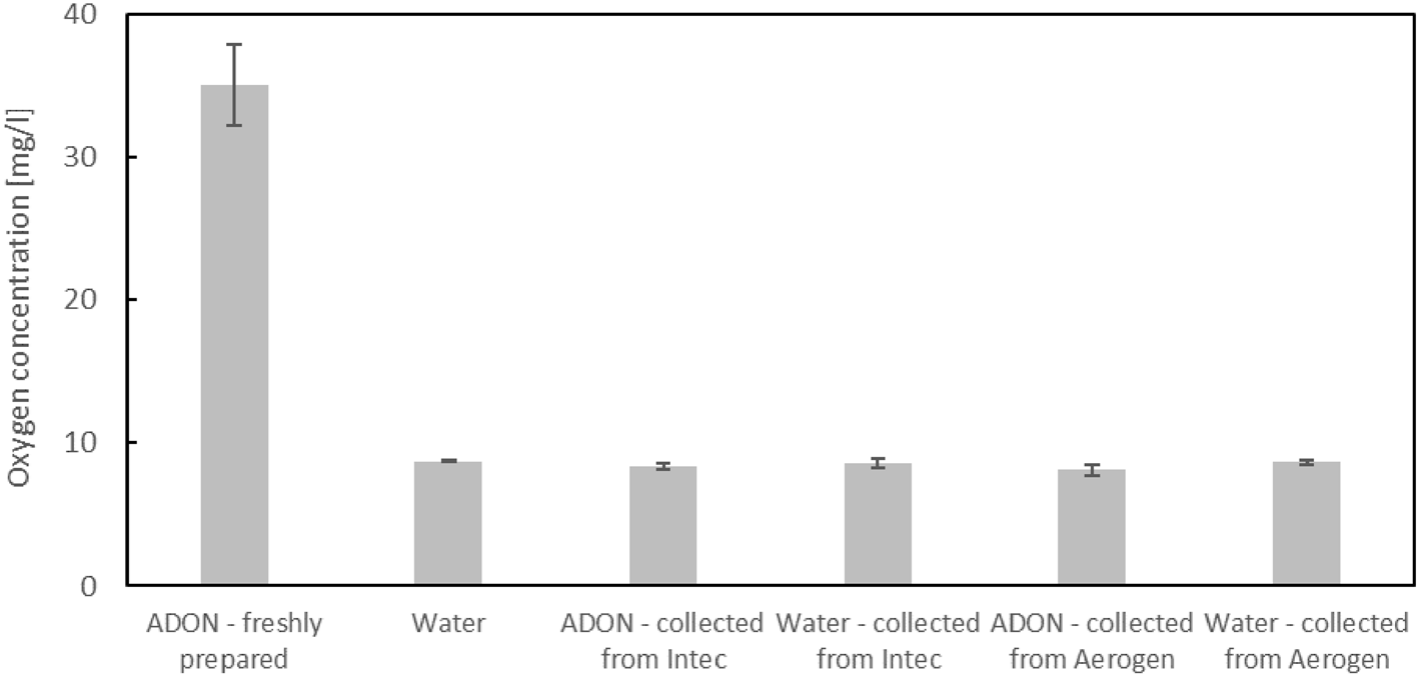
Oxygen concentration in water and ADON directly after generation and in the samples condensed after nebulization. Mean values (n = 3) + standard deviation (error bars). There is no statistically significant difference between oxygen concentration in water and samples after atomization according to post-hoc Tukey test ($\alpha = 0.05$).

Initially, we tried to measure oxygen concentration in liquids collected after nebulization by coalescence of aerosol droplets in a glass vessel. Results shown in Fig. 5 for ADON and water show similar values of oxygen concentration in the liquid phase regardless of the nebulizer used. These values were significantly lower than in the “fresh” ADON before nebulization. The results suggest that this method of analysis was ineffective, most probably due to desorption of oxygen from ADON which occurred during 20–30 min required for collection of the nebulized aerosol. Desorption process is fast because the surface area of air/water contact was very large during ADON atomization to droplets in the micrometer size-range. Under such conditions the oxygen concentration in ADON was reduced to the equilibrium value for oxygen dissolved in water contacting with air at room temperature and atmospheric pressure. However, this process does not correspond to the conditions of therapeutic application of nebulizers, where released aerosol immediately flows into the respiratory tract. In such conditions, oxygen from ADON is carried to the lungs both as NBs inside fine droplets and desorbed gaseous oxygen contained in the inhaled air. As shown before, higher oxygen concentration is preserved in ADONs during storage, ADON nebulization should increase the amount of oxygen supplied to the organism during inhalation. To demonstrate that, oxygen concentration was measured directly in mists emitted from nebulizers. The oxygen sensor used in this study could measure oxygen concentration in either air gas or liquid, but measurement in mists bear significant challenge for interpretation of obtained results due to hardship in evaluating the density of mists released. For quantitative determination of oxygen content in ADON mists, we have assumed the equilibrium concentration of dissolved oxygen in water as reference concentration for all the measurements.

Table 4 compares the oxygen content in mists emitted from VMNs for nebulized ADON and water. ADON was used directly after generation, and after 7 or 14 days of storage in securely sealed glass bottles. The columns denoted as ‘Increase [%]’ show the relative difference between the average oxygen concentrations in each sample and water. One can clearly see that oxygen content in ADON aerosol directly after nebulization, i.e., in the mist is entering the respiratory tract, was 18.85% or 13.62% higher than in the aerosol generated from water in Aerogen and Intec nebulizers, respectively. For ADONs after storage, this effect is decreased, but is still present for Aerogen. In contrast, the oxygen content in ADONs after 7 or 14 days of storage and nebulized in Intec was significantly reduced. According to post-hoc Tukey test ($\alpha = 0.05$), all of the results are significantly different from one another, beside two pairs. First pair shows no significant difference between oxygen concentration in water atomized by either Aerogen or Intec nebulizer, while the second one confirms that the oxygen concentration in ADON atomized using Aerogen nebulizer does not significantly change after 7th day of storage.

**Table 4:** Comparison of oxygen content in the mists measured directly at the outlet of VMNs for water and ADON.

Nebulized liquid	Aerogen		Intec	
	Oxygen content (mg L^−1^)	Increase (%)	Oxygen content (mg L^−1^)	Increase (%)
Water	8.72 ± 0.16	N/A	8.71 ± 0.16	N/A
ADON—freshly prepared	10.37 ± 0.18	18.85	9.91 ± 0.21	13.62
ADON—after 7 days of storage	9.46 ± 0.17	8.49	9.01 ± 0.12	3.28
ADON—after 14 days of storage	9.43 ± 0.18	8.16	8.77 ± 0.14	0.51

Values are means ± SD, n = 3.

## Discussion

Presented comprehensive studies of ADON properties show that oxygenation in the membrane system under optimized process conditions allows obtaining dispersion containing nanobubbles with the size range of 80–450 nm (count mode: 140 nm). NB size range becomes narrower due to equilibration after 1 day (range of 90–350 nm, count mode: 160 nm) and remains practically unchanged for 21 days when stored in a closed glass container at room temperature (Fig. 2, Table 3). These results confirm that it is possible to obtain stable NB dispersions for potential use in nebulization a few weeks after ADON production.

The results also show that ADONs can be effectively nebulized in various nebulizers without influencing the droplet size distribution of generated aerosol (Fig. 3). It is important factor which confirms that aerosol nebulized from ADON maintains good properties required for targeting various levels of the respiratory system. Three of the studied nebulizers (Pari, Thomex, and Aerogen) generated aerosol with the size appropriate for targeting lower airways (*Dv50* = 4.5–6 µm and FPF = 40–55%), while one nebulizer (Intec) seemed to be more useful in delivering aerosol to the upper airways (Dv50 > 12 µm). Each device's nebulization parameters were almost unchanged, regardless of the liquid used, i.e., water or ADON.

It was also confirmed that the nebulization process despite the substantial energy input to the liquid phase and the extended residence time of NBs in the device did not destroy nanobubbles, i.e., they were present in ADON collected from aerosol droplets, although NB size characteristics were modified. For instance, in pneumatic (Pari) and ultrasonic (Thomex) nebulizers, bimodal densities of number distribution functions were noted in ADON after nebulization (Fig. 4) suggesting the influence of liquid atomization mechanism on NBs present in water. However, the size characteristics of NBs in dispersions nebulized in VMNs remained stable, which allowed us to indicate these two nebulizers for further studies of oxygen content in aerosolized ADONs. It should be noted that this type of measurement was challenging since it required a few-minute period of collecting the droplets during when the liquid was contacted with the atmospheric air. Such conditions caused oxygen desorption from ADON to the air, reducing the oxygen concentration in the dispersion to the equilibrium value, as shown in Fig. 5. One may note that, conditions of this experiment which were required to evaluate oxygen content in the liquid after aerosolization do not correspond to the actual condition of nebulization when aerosol generated in the device flows directly to the respiratory system. In such a situation, the total oxygen content in the aerosol (liquid and gas phases) decides on a potential therapeutic gain from inhalation of aerosolized ADON. By doing measurements in the aerosol phase, we were able to demonstrate that the oxygen content was increased by 13.6–18.9% (depending on the VMN) for nebulized ADON compared to the nebulized distilled water (Table 4). It clearly confirms that it is possible to increase the oxygen supply during inhalation of nebulized fresh ADON. Even for ADON stored up to 2 weeks, the oxygen content in the aerosol increases above 8% for the nebulization in Aerogen VMN, although it is much lower for Intec and approaches zero for ADON after a 2-week storage time.

The results also show that not all nebulizers are suitable for ADON delivery with an effect of increased oxygen delivery during aerosol inhalation. Both jet and ultrasonic nebulizers are not recommended as the forces and processes responsible for aerosol formation (the energy of ultrasonic waves, droplet impaction, and liquid recirculation inside the nebulizing head) may significantly influence the stability of nanobubbles and result in rapid oxygen desorption during elution with high flows of auxiliary air which is used in these types of nebulizers. VMN is a better option since the aerosol is formed during a single liquid passage through the orifices in the vibrating mesh. Short contact time between ADON and air helps to keep NBs inside the droplets emerging from the nebulizer. It is also interesting to see that one VMN (Aerogen) allows obtaining a higher oxygen concentration in the aerosol phase than another (here: Intec). As shown in Fig. 3, each device produces droplets of a different size, and, intuitively, one may expect that larger droplets obtained from Intec (obviously, with a lower hydrodynamical stresses) should maintain more oxygen than finer droplets from Aerogen. However, one also may note that these nebulizers have quite different designs. The mesh material is not the same (metal in Aerogen, polymeric in Intec—Table 1), and the volume of liquid in the nebulizing vessel and the volume of air over a liquid layer are less in Aerogen than in Intec. These two factors are probably responsible for different degree of oxygen desorption during ADON nebulization, which became notable, in particular, for the samples after 1 or 2-week storage.

**Figure 6:**
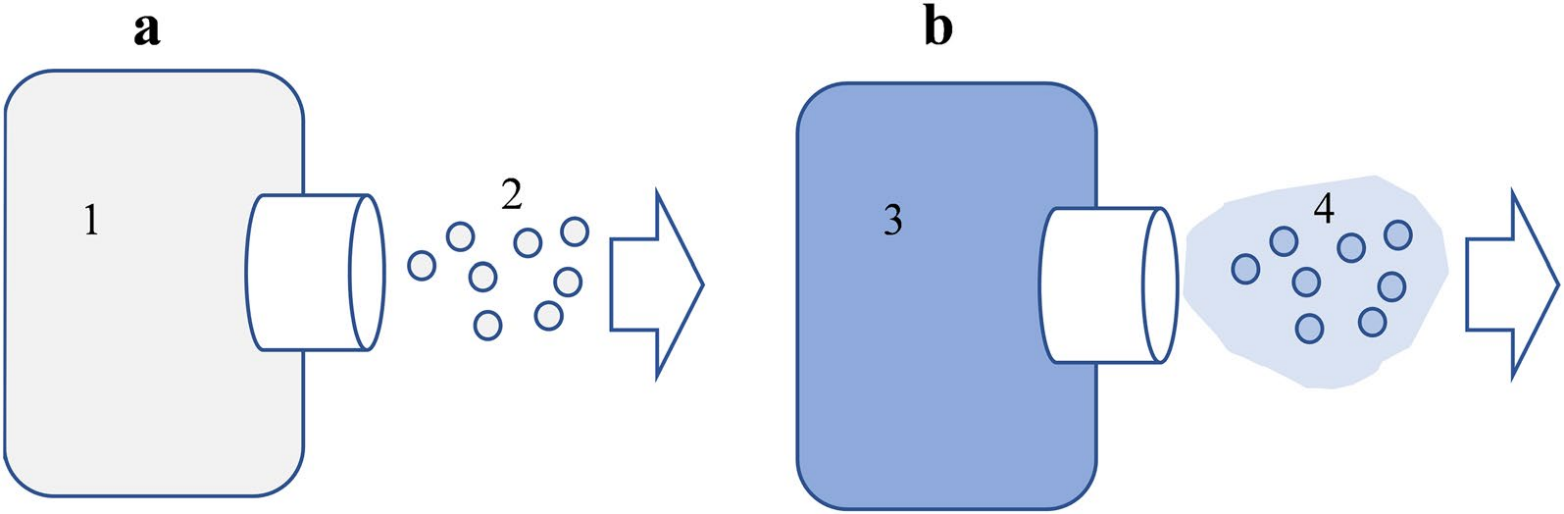
Schematic comparison of aerosol generated from (**a**) water and (**b**) ADON: 1—water in a nebulizer (with equilibrium concentration of dissolved oxygen), 2—aerosol formed by droplets of water in the air, 3—ADON (with increased oxygen concentration) in a nebulizer, 4—ADON droplets surrounded by air with increased oxygen concentration after partial desorption of this gas from ADON droplets.

It should be noted that even in the case when oxygen partially desorbs from ADON droplets nebulized in a VMN, it enriches the gas phase of the inhaled aerosol, so the total oxygen supply in the lungs is increased, as schematically shown in Fig. 6. It should be a benefit of ADON nebulization in treating pulmonary dysfunctions that are associated with reduced oxygenation or the recovery from such pathological cases.

Aqueous aerosol generated from ADON in mesh nebulizers has a higher oxygen content than water at the equilibrium conditions. It may be proposed that when ADON is used as a carrier of pulmonary medicines, treatment of lung diseases by inhalation will be enhanced by oxygen supplementation even without using of oxygen as the additional gaseous carrier. It should allow to obtain a better treatment in non-hospital conditions. The potential benefit of ADON as a new drug carrier is the decreased time spent in the hospital, hence the reduced costs (both economic and psychological). Inhalation of nebulized ADON may also be suggested as a method of home-based pulmonary recovery and rehabilitation after hospital treatment of severe pulmonary dysfunctions, e.g., caused by COVID-19. The above analysis is based purely on physicochemical considerations, which is a limitation of our study, so future applications of aqueous dispersions of oxygen nanobubbles needs in vivo studies to confirm the proposed pharmacological effects.

## Conclusions

We proposed a new potential application area for liquid dispersions of oxygen nanobubbles which dynamically gain approval in the multiple branches of science and industry. Obtained results indicate their potential usefulness also in the treatment of respiratory diseases by inhalation of aerosols. The increased oxygen content in the aerosol droplets generated in two mesh nebulizers suggests that the proposed concept may be helpful in the oxygen supplementation during drug delivery by aerosol inhalation without using an additional oxygen source. This application can increase the overall effectiveness of lung disease treatment and pulmonary rehabilitation, simultaneously reducing social and economic costs. Besides in vivo studies that are needed to confirm the expected oxygenation effect, the next research steps should be focused on the determination of the interactions of ADONS with various inhalation drugs considering also the effect on the nanobubble size and stability in such mixtures.

## Data Availability

The datasets generated and analyzed during the current study are available from the corresponding author on reasonable request.
